# Roboterassistierte Chirurgie im Kopf-Hals-Bereich

**DOI:** 10.1007/s00106-026-01742-4

**Published:** 2026-02-24

**Authors:** Chia-Jung Busch, Tilman Huppertz, Markus Blaurock, Christopher Seifen, Lukas Wittig, Benjamin Fenske, Balazs B. Lörincz, Christian Betz

**Affiliations:** 1 Klinik und Poliklinik für Hals‑, Nasen, Ohrenkrankheiten, Kopf- und Halschirurgie, Universitätsmedizin Greifswald, Fleischmannstr. 10, 17475 Greifswald, Deutschland; 2 Hals‑, Nasen‑, Ohrenklinik und Poliklinik, Universitätsmedizin Mainz, Langenbeckstraße 1, 55131 Mainz, Deutschland; 3 Klinik und Poliklinik für Hals‑, Nasen‑, Ohrenheilkunde, Universitätsklinikum Hamburg- Eppendorf, Martinistr. 52, 20246 Hamburg, Deutschland; 4 Kopf-Hals-Klinik für HNO-Heilkunde und Onkologische, Rekonstruktive, Endokrine und Roboter-assistierte Kopf-Hals-Chirurgie der Agaplesion Frankfurter Diakonie Kliniken, Bethanien und Markus Krankenhäuser, Frankfurt am Main, Deutschland

**Keywords:** Robotische Chirurgie, Oropharynxtumoren, Kopf-Hals-Tumoren, Chirurgische Onkologie, Minimal invasive Chirurgie, Robotic surgery, Oropharyngeal neoplasms, Head and neck neoplasms, Surgical oncology, Minimally invasive surgical procedures

## Abstract

Die roboterassistierte Chirurgie hat sich im Kopf-Hals-Bereich, insbesondere bei Tumoren des Oropharynx, als vielversprechende minimal invasive Methode etabliert. Sie ermöglicht präzise, gewebeschonende Eingriffe mit guter onkologischer Kontrolle und funktionellem Erhalt. Moderne Systeme bieten Multi-Port- und Single-Port-Technologien, die je nach Anatomie und Indikation unterschiedliche Vorteile aufweisen. Transorale Zugänge erlauben eine narbenfreie Resektion mit verkürztem Heilungsverlauf. Trotz wachsender Evidenz bleibt die roboterassistierte Chirurgie kostenintensiv und technisch anspruchsvoll. Ausbildung, Fallzahlen und Zugang zur Technologie sind limitierende Faktoren. Zukünftige Entwicklungen wie die Integration von künstlicher Intelligenz (KI) und verbesserte Visualisierung versprechen weitere Optimierungen. Der vorliegende Artikel vermittelt aktuelles, praxisrelevantes Wissen zur Indikationsstellung, Technik und klinischen Bewertung roboterassistierter Verfahren im Kopf-Hals-Bereich.

## Abstract (en)

Robot-assisted surgery has been established in the head and neck region as a promising minimally invasive method, particularly for oropharyngeal tumors. It enables precise tissue-preserving procedures with good oncological control and functional preservation. Modern systems offer multi- and single-port technologies, each providing distinct advantages depending on anatomy and indication. Transoral approaches allow scar-free resection with shortened recovery times. Despite growing evidence, robot-assisted surgery remains cost-intensive and technically demanding. Training requirements, case numbers, and access to technology are limiting factors. Future developments such as AI integration and enhanced visualization promise further improvements. This article provides up-to-date practice-oriented knowledge on indications, techniques, and clinical evaluation of robot-assisted procedures in the head and neck region.

Die roboterassistierte Chirurgie hat in den letzten Jahren erheblich an Bedeutung gewonnen und eröffnet auch im Kopf-Hals-Bereich neue therapeutische Möglichkeiten. Insbesondere bei Oropharynxtumoren bietet sie präzise, funktionserhaltende Eingriffe mit minimaler Invasivität. Der vorliegende Artikel ist für die klinische Praxis relevant, da er aktuelle Indikationen, Techniken, Ergebnisse und zukünftige Entwicklungen systematisch darstellt.

## Grundlagen der roboterassistierten Chirurgie

### Prinzipien und Funktionsweise chirurgischer Robotersysteme

Aktuell setzt die roboterassistierte Chirurgie ein Master-Slave-System voraus, bei dem der Operateur die vollständige Kontrolle und die komplette Verantwortung übernimmt. Die unterschiedlichen chirurgischen Instrumente werden in die Roboterarme geladen, die von der Steuerkonsole durch den Chirurgen bedient werden. Der wichtigste Vorteil, der „single most important selling point“ eines roboterassistierten chirurgischen Systems ist das Vorhandensein der Handgelenke an den Instrumenten. Andere minimal invasive Verfahren, wie die Laparoskopie im Bauch bzw. im Becken oder die transorale Laser-Mikrochirurgie (TLM) in der Kopf-Hals-Chirurgie, verfügen über kein Handgelenk am Ende der schneidenden oder greifenden Instrumente, daher können sie nur gerade, nach vorne schneiden oder präparieren, ohne laterale Freiheitsgrade. Glasfaser oder Hollow-Conduit-Systeme in der Laserchirurgie konnten sich wegen des relativ hohen Energieverlusts und der immer noch deutlich eingeschränkten lateralen Beweglichkeit nicht wirklich durchsetzen. Im Kopf-Hals-Bereich besitzt aktuell ausschließlich das daVinci®-System (Fa. Intuitive, Sunnyvale, CA, USA) klinische Relevanz, da alle anderen robotischen Plattformen entweder gescheitert oder nicht zugelassen sind. Fa. MedRobotics (Raynham, MA, USA) und Fa. CMR Surgical (Cambridge, Vereinigtes Königreich) sind mittlerweile insolvent, das Hugo-System von Fa. Medtronic (Meerbusch, Deutschland) ist für den Kopf-Hals-Bereich weder in den USA noch in Europa zugelassen und verfügt über keine Single-Port(SP)-Variante, und Fa. Titan Medical (Toronto, Kanada) hat nie ein marktreifes Produkt entwickelt. Somit ist das daVinci®-System derzeit das einzige verfügbare und klinisch etablierte Robotiksystem in unserem Fachgebiet.

### daVinci®-System

**Abb. 1:**
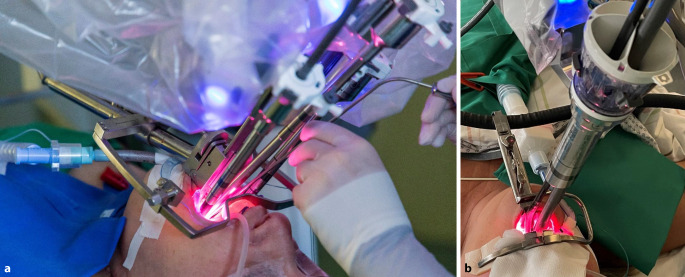
Vergleich Multi-Port-System (**a**) zu Single-Port-System (**b**)

Das weltweit am meisten verbreitete System, das daVinci®-System (Fa. Intuitive, Sunnyvale, CA, USA), hat bisher 5 Generationen. Die aktuelle vierte Generation umfasst das X und Xi als Multi-Port-Systeme, und das SP als Single-Port(SP)-System (Abb. 1). Von der fünften Generation wurde bisher nur das daVinci‑5 als Multi-Port-System auf den Markt gebracht; das dazu gehörende SP System befindet sich noch in der Entwicklungsphase.

#### Hauptkomponenten

Alle Systeme bestehen hauptsächlich aus 3 Hauptkomponenten: „patient cart“ (Patientenwagen), „vision cart“ (Video- und Geräteturm) und „surgeon console“ (Operateur-Konsole). Die chirurgischen Instrumente und das Endoskop werden in die Arme des „patient cart“ geladen und vom Operateur über die „surgeon console“ gesteuert. Die Instrumente und die Endoskope sind zwischen den X- und Xi-Systemen umtauschbar, nur das SP hat eigene Instrumente und eine flexible „Snake-Kamera“.

#### Weitere technische Komponenten und Instrumente

Es gibt zahlreiche gelenkte, sog. EndoWrist-Instrumente für das daVinci®-System, wovon 5 Typen – 2 monopolare und 3 bipolare Instrumente – für transorale roboterassistierte Chirurgie („transoral robotic surgery“, TORS) besonders geeignet sind. Als monopolares Schneideinstrument ist der monopolare Spatel für den Oropharynx [14, 15] und die monopolare Schere für den Hypopharynx und den supraglottischen Kehlkopf zu empfehlen [11, 15]. Als bipolares Greifinstrument ist die Maryland-Pinzette für den oberen Oropharynx, die Fenestrated-Bipolarpinzette für den Zungengrund, und die Curved-Bipolarpinzette für den Hypopharynx und den supraglottischen Kehlkopf zu empfehlen. Für die Wahl zwischen der 0°-Optik und der 30°-Optik (nur bei Multi-Port-Systemen) gilt folgende Faustregel: Soweit möglich, ist die 0°-Optik zu bevorzugen, weil sie eine größere Bewegungsfreiheit für die Instrumente ermöglicht. Bei zahnlosen Patienten kann die 0°-Optik bis in die Vallecula eingesetzt werden, bei einzelnen Fällen sogar bis in die Supraglottis [13]. Beim Docken ist das Fernsteuerungszentrum („remote center“; Drehpunkt, „pivot point“) der Instrumente auf dem Niveau der Mundöffnung zu positionieren; dagegen sollte der Punkt „remote center“ der Kamera etwa 2–3 cm außerhalb der Mundöffnung positioniert werden, damit eine häufige Kollision der Trichter der Trokare vermieden wird. Was die Energie-Einstellungen angeht, gilt folgende Faustregel: je niedriger, desto besser – bei möglichst hoher Gewebespannung („traction/counter-traction“). Da die monopolare Schneidetechnik hauptsächlich zur angelsächsischen Schule gehört und viele deutsche bzw. kontinentale Kopf-Hals-Chirurgen früher wenig bis keine Erfahrung mit der monopolaren Schneidetechnik hatten, stellt ihre TORS-Lernkurve gleichzeitig die monopolare Lernkurve dar, wobei die richtige Energie-Einstellung eine entscheidende Rolle spielt [13]. Ein sehr häufiger Fehler bei TORS-Anfängern ist die Benutzung zu hoher Watt-Stärken (> 50 W), was die Rate an Blutungen, Ödemen und insgesamt die Rate der Kollateralschäden deutlich erhöht.

## Transorale roboterassistierte Chirurgie im Kopf-Hals-Bereich

### Indikationen

Prinzipiell ähneln die Indikationen für TORS denen anderer transoraler Resektionsverfahren. Allerdings ergeben sich einerseits deutliche Vorteile durch die Abwinkelbarkeit der Kamera sowie der Instrumente, die das Spektrum erweitert haben. Andererseits ist die Exposition der Befunde durch die Kombination verfügbarer Retraktoren und die Sperrigkeit der Roboterarme insbesondere bei Multi-Port-Systemen teilweise behindert.

Bei der Indikationsstellung zur TORS sind generell folgende Faktoren zu berücksichtigen:

- transorale Einstellbarkeit,
- Operabilität des Patienten,
- Zulassungsbeschränkungen der Systeme.

Die transorale Einstellbarkeit sollte – bestenfalls mittels Panendoskopie eruiert – für TORS adäquat sein. Weitere Indikatoren, die prädiktiv hinsichtlich der Einstellbarkeit sein können, sind beispielsweise der modifizierte Mallampati-Score [16] oder der Interdentalabstand bei maximaler Mundöffnung. Dabei ist zu berücksichtigen, dass auch die Einführbarkeit und die Beweglichkeit der Gerätschaften mit einkalkuliert werden müssen. Hierbei unerlässlich sind ein adäquates, systematisches Training sowie hinreichende Erfahrungswerte.

Neben generellen Faktoren spielen bei der Operabilität des Patienten insbesondere Antikoagulanzien eine Rolle, für die eine signifikant erhöhte Nachblutungsgefahr beschrieben wurde [18]. Acetylsalicylsäure (ASS) in kardioprotektiver Dosierung scheint hier allerdings eine Ausnahme darzustellen, und von einer kontinuierlichen Einnahme wird derzeit nicht mehr abgeraten [1].

Zugelassene Indikationen können durchaus von System zu System sowie von Land zu Land divergieren. Zulassungsbeschränkungen der Systeme haben selbstredend Einfluss auf die Indikationsstellung.

Derzeit (Stand 09/25) bestehen für TORS in Europa vornehmlich folgende Indikationen:

- Malignome wie Oropharynxkarzinome,
- benigne Erkrankungen wie schlafbezogener Atemstörungen.

Oropharynxkarzinome machen national und international den Hauptteil der Indikationen aus, wobei insbesondere exophytisch wachsende Tonsillen- und Zungengrundkarzinome im Stadium T1–2 zumeist für TORS geeignet sind. In geringerem Umfang können sich auch kleinere Hypopharynx- und supraglottische Larynxkarzinome eignen. Prinzipiell sprechen folgende, tumorbezogene Faktoren eher gegen TORS: Mittellinienbezug, Infiltration größerer Anteile des Weichgaumens (falls hier keine Rekonstruktion beispielsweise über Buccinatorlappen stattfinden kann), Beteiligung des parapharyngealen Fettgewebes, Angrenzen an Zungenbein/Kehlkopf, Infiltration der extrinsischen Zungenmuskulatur [4]. Des Weiteren sollte beachtet werden, dass die Vorteile von TORS vor allem zu Tragen kommen, wenn *keine *postoperative, adjuvante Radio(chemo)therapie notwendig ist. Demzufolge sollte TORS eher nicht indiziert werden, falls schon prätherapeutisch eindeutige Indikatoren dafür vorliegen, dass dies notwendig wird [5]. Eine Sonderstellung nimmt die Abtragung der Zugengrundtonsille im Rahmen der diagnostisch-therapeutischen Behandlung zervikaler CUP-Syndrome („cancer of unknown primary“) ein, die in Kombination mit der beidseitigen Tonsillektomie durchgeführt werden kann und für die – v. a. für auf humane Papillomaviren (HPV) positive Lymphknotenmetastasen – eine hohe Tumordetektionsrate gezeigt wurde [3]. Inwiefern weitere sinnvolle Indikationen für die TORS bei Kopf-Hals-Malignomen bestehen – beispielsweise für die Resektion von Oropharynxkarzinomen im Stadium T3–4 in Verbindung mit Rekonstruktionen oder für die transorale Laryngektomie als Salvage-Option bei Rezidivlarynxkarzinomen (zur Reduktion der postoperativen Fistelrate) – ist anhand der vorliegenden Literatur derzeit noch nicht abschließend beurteilbar.

Benigne Erkrankungen: Hier stehen v. a. Eingriffe zur Behandlung schlafbezogener Atemstörungen im Vordergrund. Bei guter Indikationsstellung –bestenfalls mittels DISE („drug-induced sleep endoscopy“)– scheint die Abtragung einer hyperplastischen Zungengrundtonsille zu einer signifikanten Verbesserung entsprechender polysomnographischer Parameter zu führen [2]. Abgesehen davon bestehen im Kopf-Hals-Bereich auch noch Indikationen zu „narbenfreien“ Resektionen bei Schilddrüsenerkrankungen über einen transaxillär-transmammären Zugang („bilateral axillo-breast approach robotic thyroidectomy“, BABART) oder transoralen Zugang („transoral robotic thyroidectomy“, TORT) und auch teilweise Neck-Dissections. Diese werden vornehmlich im asiatischen Raum durchgeführt. Die Mehrzahl der Publikationen zu der Thematik stammt ebenfalls aus dieser Region. Obgleich es an randomisierten Vergleichsstudien mangelt, erscheinen die Methoden – bei vergleichsweise erhöhter Op.-Dauer – sicher und vom Ergebnis vergleichbar mit offenen Verfahren [6].

### Praktische Aspekte bei der Durchführung

Die korrekte Lagerung stellt eine wesentliche Voraussetzung für erfolgreiche TORS dar. Grundlage ist eine stabile Rückenlage, wobei der Schultergürtel in neutraler Position bleibt und die oberen Extremitäten seitlich angelagert werden. Der Kopf sollte – abhängig von der zervikalen Mobilität – in eine leichte Reklination (15–30° Extension) gebracht werden. Dies optimiert die Exposition des Op.-Felds und erleichtert die Stabilisierung des Retraktorsystems sowie die Instrumentenführung.

Bei Resektionen im Bereich des Zungengrunds, Hypopharynx oder Larynx erfolgt die Lagerung bei einer Tischposition von 0°. Im Gegensatz dazu ist bei der lateralen Oropharyngektomie eine 15°-Kopftieflage zu bevorzugen.

#### Einsatz von Mundsperrern

Die Wahl des Retraktors richtet sich nach Tumorlokalisation und anatomischen Gegebenheiten:

- Lateraler Oropharynx: McIvor- oder Crowe-Davis-Sperrer
- Zungengrund, Hypopharynx, Larynx: Feyh-Kastenbauer-Sperrer (FK-Sperrer; Fa. Olympus, Tokio, Japan) bzw. LARS‑2.0‑Retraktor (Fa. Fentex, Neuhausen ob Eck, Deutschland)

Für den Zugengrund bewährt hat sich der Feyh-Kastenbauer-Sperrer (FK-Sperrer; Fa. Olympus) bzw. LARS‑2.0‑Retraktor (Fa. Fentex), wobei Ersterer bedauerlicherweise nicht mehr verfügbar ist. Beim Einsatz dieser Sperrer ist darauf zu achten, die Zahnreihe des Oberkiefers sowie die Zunge und Unterlippe ausreichen zu schützen, um hier Verletzungen zu vermeiden.

#### Schutzmaßnahmen und Intubationsweg

Zum Schutz des Patienten wird empfohlen, Augenprotektoren anzulegen. Die Wahl des Intubationsverfahrens richtet sich nach dem jeweiligen Eingriffstyp. Bei Zungengrundresektionen und der lateralen Oropharyngektomie wird i. d. R. eine nasale Intubation bevorzugt. Bei Hypopharynx- und Larynxeingriffen wird eine Tracheotomie empfohlen, um die Exposition bestmöglich zu erleichtern.

Während des Andockens des Robotersystems muss die Kopfposition unbedingt stabil bleiben, um ein Verschieben des Op.-Felds oder der Instrumente zu verhindern.

#### Roboter-Docking und Raumanordnung im Operationssaal

Die Raumanordnung und das strukturierte Docking des Op.-Roboters sind entscheidend für einen reibungslosen Ablauf. Das Andocken des Robotersystems erfolgt i. d. R. seitlich zum Patienten, wobei der Op.-Tisch so niedrig wie möglich positioniert wird. Dies gewährleistet ausreichend Bewegungsfreiheit für die Roboterarme.

Die chirurgische Konsole befindet sich außerhalb des sterilen Bereichs bzw. des Instrumententischs, während ein Assistent am Op.-Tisch positioniert ist, um bei Bedarf mit konventionellen Instrumenten zu unterstützen. Eine klare Trennung von Zonen zwischen den Instrumentenarmen, Instrumententisch sowie freier Zugang zum Mundraum sind essenziell für eine sichere Durchführung des Eingriffs.

Vor dem Docking ist darauf zu achten, dass Op.-Leuchten und nicht benötigtes Equipment zur Seite geschoben werden. Zudem muss die Lagerung abgeschlossen und der Sperrer fixiert sein. Das spezifische Docking hängt maßgeblich vom Modell des Roboters, des Op.-Tischs und der Anordnung im Op.-Saal ab.

#### Grundlage des operativen Vorgehens

Bei der Einstellung der Instrumente sollte ein Effekt von 2 mit einer maximalen Leistung von 35–40 W bevorzugt bei „swift coag“ und „dry cut“ auf keinen Fall überschritten werden. Die grundlegenden Op.-Schritte können beispielsweise der Publikation von Holsinger entnommen werden [4]. Das Resektionsausmaß sollte am Anfang der Operation klar definiert werden. Die anatomischen Strukturen des parapharyngealen Raums sollten gut bekannt sein, damit bei der Resektion von Entitäten im Bereich der lateralen Pharynxwand die vaskulären Strukturen rechtzeitig identifiziert, geschont oder ggf. abgesetzt werden können. Das parapharyngeale Fett und die Konstriktormuskeln sind nach lateral und zur Tiefe prognostisch relevante Strukturen, die leicht zu identifizieren sind. Je nach Invasionstiefe ist es ggf. notwendig, die Mm. styloglossus und stylopharyngeus mit zu resezieren. Die A. carotis interna liegt zwischen beiden Muskeln in der Tiefe, wird aber i. d. R. bei der Präparation nicht dargestellt.

Das vorrangige Ziel ist das Erreichen einer R0-Resektion. Insbesondere junge Chirurgen mit wenig Erfahrung bei roboterassistierten Operationen sollten zunächst sehr kritisch ihre Fälle auswählen und bekannte Kontraindikation, z. B. radiologischer Art, beachten [10].

## Roboterassistierte Chirurgie im Vergleich zu konventionellen Verfahren

Der Einsatz von Roboterchirurgie ist bislang im Vergleich zur konventionellen Chirurgie noch wenig erforscht. Roselló und Lai vergleichen in systematischen Reviews die offene und endoskopische Chirurgie mit der Roboterchirurgie [8, 17].

Im Vergleich zur offenen Chirurgie konnten durch Roselló 4 Publikationen mit 371 Patienten ausgewertet werden, wobei der Fokus hier auf der Behandlung von Oropharynxkarzinomen lag. Bezüglich der onkologischen Sicherheit gab es keinen signifikanten Unterschied, mit Ausnahme einer Publikation zugunsten TORS. Postoperative Blutungen wurden bei TORS seltener beobachtet, bei vergleichbarer Fistelrate. Die Krankenhausaufenthaltsdauer war bei TORS geringer, eine Dekanülierung gelang früher. Die Schluckrehabilitation war bei TORS früher möglich.

Lai et al. verglichen die Roboterchirurgie mit der endoskopischen Laserchirurgie mit Fokus auf den Hypopharynx. Hierbei wurden 8 TORS-Studien eingeschlossen, die insgesamt 147 Patienten umfassten, sowie 3 Studien zur endoskopischen Laserchirurgie mit 139 Patienten [8]. Beim Vergleich der Komplikationen fand sich für die Blutungsrate kein signifikanter Unterschied; die Rate von Aspirationspneumonien war in der Laserkohorte signifikant größer. Die Notwendigkeit einer perkutanen endoskopischen Gastrostomie (PEG) bei TORS bestand in unter 5 % der Fälle, und eine langfristige Tracheotomie war in unter 2,5 % der Fälle erforderlich. Aufgrund der weitestgehend retrospektiven Analysen ohne randomisierte Studien bleibt der direkte Vergleich mit konventionellen Techniken limitiert.

## Limitationen

### Technische und anatomische Einschränkungen

Anatomische Einschränkungen sind nicht speziell auf das daVinci®-Operationssystem bezogen, sondern auf allgemeine Merkmale der transoralen Chirurgie wie Einstellbarkeit des Op.-Felds, Kopfreklination oder Adipositas. Diese Punkte sollten wie beschrieben vor dem Eingriff sicher evaluiert werden. Hinzu kommt, insbesondere für das Multi-Port-System, dass die Robotersysteme primär nicht für den kleinen Kopf-Hals-Markt konzipiert wurden und daher die Handhabbarkeit der Instrumente im Bereich der Mundhöhle und des Pharynx oft nicht optimal ist.

### Kosten und Verfügbarkeit

Hierunter fällt zunächst die Verfügbarkeit des daVinci®-Operationssystems mit Anschaffungskosten von etwa 3 Mio. €. Zudem besteht die Notwendigkeit einer interdisziplinären Koordination der beteiligten Fachabteilungen (Allgemeinchirurgie, HNO-Heilkunde, Urologie, Thoraxchirurgie, Gynäkologie) hinsichtlich Op.-Dauern, Kapazitäten der Anästhesie und Op.-Pflege. Nur so wird eine bestmögliche Auslastung der Geräte erreicht und die Wirtschaftlichkeit garantiert.

Die hohen Kosten der Erstanschaffung müssen sich im Laufe der Nutzungsdauer amortisieren. Dies kann nur in operativen Zentren mit entsprechend großen Fachabteilungen gewährleistet werden. Zudem gibt es laufende Kosten für Abdeckungen, Einmalmaterial und für die wiederverwendbaren Arme des Operationssystems. Diese liegen für Kopf-Hals-Eingriffe bei Verwendung von 2 Instrumenten bei etwa 1050 € und bei Verwendung von 3 Instrumenten bei etwa 1300 € entsprechend den Angaben des Herstellers.

### Ausbildungs- und Lernkurvenproblematik

Eine der wichtigsten Limitationen ist die vergleichsweise aufwendige und kostenintensive Ausbildung der Operateure und des Pflegepersonals. Die Ausbildung erfolgt über den Hersteller, wobei vor Durchführung der ersten Operation ein Lern- und Anwendungsmodul mit zunächst mehrtägiger Hospitation bei erfahrenen Anwendern, gefolgt von einem simulierten Training an den Op.-Konsolen, durchgeführt wird. Dem schließt sich der Besuch eines Trainingszentrums mit intensiver Einführung in Geräteaufbau und -funktionen sowie Probeoperationen an Tiermodellen an. Parallel erfolgt die Schulung der Op.-Pflege an Geräteaufbau, -abdeckung, -nutzung und -abbau sowie Aufbereitung der sterilisierbaren Teile des Instrumentariums. Die ersten Operationen erfolgen unter Supervision erfahrener Proctoren und Betreuung durch Personal des Herstellers. Auch bei anschließenden Eingriffen sind Vertreter des Herstellers anwesend, um einen reibungslosen Ablauf der Operationen zu gewährleisten. Dabei gibt es eine deutliche Lernkurve bei Operateur, Tischassistenz, Op.-Pflege und Anästhesie mit anfänglich längerer Op.-Dauer und Saalnutzungsdauer, die bei regelmäßiger Nutzung den Schnitt-Naht-Zeiten bei konventionellem Vorgehen ähnelt [12].

## Herausforderungen und Ausblick

Die roboterassistierte Chirurgie im Kopf-Hals-Bereich erlebt derzeit einen deutlichen Innovationsschub. Neue Systeme und Technologien eröffnen vielfältige Möglichkeiten für präzisere, schonendere und individuell zugeschnittene Eingriffe. Dabei stehen insbesondere neuartige Robotikplattformen, künstliche Intelligenz (KI), Bildnavigation sowie minimal invasive Zugänge im Fokus der Entwicklung.

Trotz technologischer Fortschritte bestehen weiterhin Herausforderungen in der klinischen Integration, insbesondere hinsichtlich Kosten, Zugangsmöglichkeiten und Ausbildung. Dennoch zeigen Umfragen unter jungen europäischen HNO-Chirurgen eine zunehmende Akzeptanz und ein wachsendes Interesse an roboterassistierten Verfahren [9]. Zukünftige Entwicklungen dürften sich verstärkt auf benutzerfreundlichere Systeme, KI-gestützte Op.-Assistenz sowie eine erweiterte Indikationsstellung fokussieren [7].

## Fazit für die Praxis

- Roboterassistierte Chirurgie erweitert die therapeutischen Möglichkeiten im Kopf-Hals-Bereich.
- Unterschiede zwischen Multi-Port- und Single-Port-Systemen sollten bei der Op.-Planung berücksichtigt werden.
- Transorale Roboterchirurgie (TORS) ermöglicht präzise, gewebeschonende Resektionen mit funktionellem Erhalt.
- Eine sorgfältige Indikationsstellung sowie Kenntnis von Chancen und Limitationen sind für die Praxis entscheidend.
- Zukünftige Entwicklungen wie die Integration von künstlicher Intelligenz (KI) und verbesserte Visualisierung versprechen zusätzliche Optimierungen.
